# SPRINTR: Swedish PRecision medicine Initiative for Novel Treatments and Research-towards efficient recruitment to clinical trials for prostate cancer

**DOI:** 10.2340/ao.v65.45126

**Published:** 2026-05-04

**Authors:** Karin Welén, Andreas Josefsson

**Affiliations:** a Institute of Clinical Sciences, Sahlgrenska Academy, University of Gothenburg, Gothenburg, Sweden; b Department of Diagnostics and Intervention, Umeå University, Umeå, Sweden

**Keywords:** Precision medicine, prostatic neoplasms, gene expression profiling, genetic profile, molecular imaging, patient recruitment

## Introduction

Precision medicine is key to maximizing the benefits of advanced therapies while reducing costs and side effect moving from risk stratification based on few clinical parameters to ever smaller molecularly defined patient subgroups. This sub-categorization requires screening larger populations to identify cohorts for biomarker-driven trials, necessitating multicenter, structured, clinically integrated workflows for recruitment and biomarker profiling.

Rapid technological advances have enabled development of cancer-specific gene panels, broader RNA sequencing, proteomics and metabolomics, and spatial mapping of -omics and tumor microenvironment at single-cell resolution, greatly advancing the deciphering of signalling pathways and biomarkers [1]. Molecular profiling has still not been integrated into routine diagnostics for prostate cancer, even though profiling efforts show prognostic and/or treatment-predictive potential [2–11].

SPRINTR (Swedish PRecision medicine Initiative for Novel Treatments and Research) is a national research infrastructure for prostate cancer precision medicine with a prospective observational cohort under a broad ethical approval as its backbone. It creates a study-ready population covering research on biomarkers, quality of life, and health economics related to prostate cancer diagnosis and treatment. The SPRINTR concept could be scalable for other cancer forms to facilitate efficient cancer precision medicine trials.

## Patients/material and methods

### Study design

SPRINTR is designed as a national research infrastructure and enabling platform rather than a fixed clinical study protocol, allowing adaptive integration of biomarker technologies over time. The purpose is to enable multiple studies addressing distinct research questions related to diagnosis, prognosis, treatment prediction, health economics, and quality of life. The SPRINTR study (2024-02263-01 and 2026-01190-01) ethical approval covers research on identifying, comparing, developing, and validating biomarker tools for prostate cancer diagnosis and treatment, as well as co-morbidities, quality of life, and health economic parameters. Participation requires informed consent to be registered in the study database and does not involve biopsies or additional study-specific procedures. Consent includes biomarker profiling of routine tissue samples and optional biobanking of blood and urine. Participants agree to the use of all clinical and research data for trial eligibility matching and centralized invitations. After inclusion, they may have no further contact or be invited to additional studies or trials requiring separate consent.

### Procedures

National participation is important, and sites will be allowed to participate at differing levels. At a minimum this means integration of procedures for information, study invitation and registration of informed consents in medical records. There are no specific exclusion criteria except inability to provide informed consent. All patients entering the diagnostic workflow are therefore eligible, allowing invitation to participate to be systematically integrated into the diagnostic care pathway at participating sites. This enables standardized, low-burden patient inclusion as part of routine clinical workflow without requiring case-by-case eligibility decisions by clinical personnel. An electronic signing module with a unique study-ID connected to the PIN is being implemented. Study staff annotates the consent in medical records. Men not receiving a biopsy-confirmed prostate cancer diagnosis remain registered in the cohort. Baseline data are retained, with limited follow-up restricted to vital status and any subsequent cancer diagnosis.

A national real-time omics workflow is being implemented in clinical pathology. During standard diagnostic prostate biopsy processing, predefined tissue allocation pathways allow parallel routing of biopsy material for research purposes in addition routine diagnostic procedures. This enables immediate access to representative tissue fractions for the SPRINTR infrastructure. Plasma, buffy coat, and the first portion of urine can be sampled utilizing standardized sampling and freezing protocols through the standard hospital referral systems. Biological samples are handled within the national biobank infrastructure (Biobank Sweden) under a central biobank agreement applicable to all participating regions.

The biomarker workflow builds on this integrated sampling framework, and protocols will be continuously evaluated and updated. Initially, diagnostic biopsy material will undergo H&E staining and immunohistochemical staining for Ki-67, PSA and PTEN, with centralized slide scanning to ensure standardized digital images suitable for AI-based image analysis. In parallel, designated tissue fractions are processed for DNA and RNA extraction, followed by genomic analysis using the GMS560 sequencing panel (Genomic Medicine Sweden [12]) and RNA sequencing for classification according to established models [2, 7, 9–11, 13].

### SPRINTR as an open research platform

**Figure 1:**
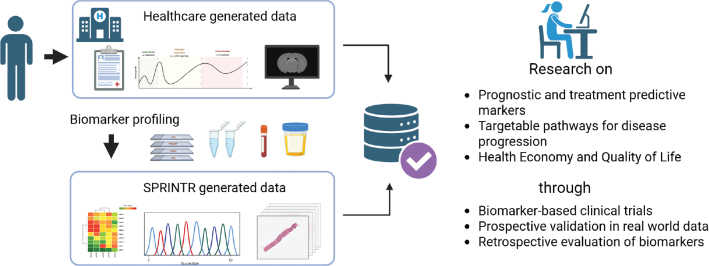
Illustration of the SPRINTR-Real research concept. By accepting participation, a person gives SPRINTR access to healthcare data from medical records, diagnostic images, quality registers and national health related registers, as well as all generated biomarker data from tissue, blood, and urine. Research questions are regulated by a broad ethical permission, including prognostic and predictive biomarkers, co-morbidities, disease progression, quality of life, and health economics. Data include lab parameters, evaluation of physical examinations, magnetic resonance imaging (MRI) and positron emission tomography (PET) imaging as well as regional quality registers and national registers on diagnoses, prescribed drugs, and causes of death. Created in https://BioRender.com

SPRINTR utilizes centralized data retrieval from national and regional healthcare and quality registers, minimizing manual data entry at participating sites (Figure 1). No study-specific eCRF is required. SPRINTR integrates standardized clinical and registry-based data with molecular data generated through national genomic medicine platforms, including sequencing- and transcriptomics-based profiling of clinical samples. SPRINTR has steering group comprising representatives from all seven medical universities in Sweden and their regional counterparts as well as the Swedish patient organization (Prostatacancerförbundet). It serves as the central coordinating steering group responsible for study approvals, data access, and strategic prioritization, ensuring compliance with ethical permits and transparency for both academic and commercial research proposals. Researchers can submit study proposals to the steering group. Approved studies need to adhere to the open research practices and make the generated data accessible to others.

**Figure 2:**
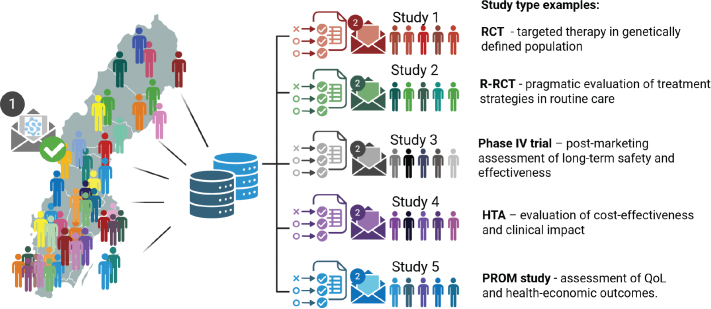
Illustration of the SPRINTR-Real concept for a study-ready population and study examples. Nation-wide invitation (1) and recruitment create a broad dynamic growing well-documented population with automatic follow-up. Built-in modules for selection on inclusion/exclusion criteria enable selected invitations (2) to generate cohorts for different types of studies, within the SPRINTR ethical approval or as add-on trials with separate regulatory frameworks. RCT: randomized controlled trial; R-RCT: register-randomized trial; Phase IV: post-marketing trial; HTA: health technology assessment; QoL: quality of life; PROM: patient-reported outcome measurement. Created in https://BioRender.com

SPRINTR aims to facilitate and increase the number of biomarker-driven clinical trials in Sweden. Importantly, all participants agree to be contacted for recruitment into other studies or trials based on information in the database, such as genetic alterations, medications, or comorbidities; this feature creates a study-ready population that facilitates nationwide recruitment (Figure 2). The inclusion started at Norrland’s University Hospital in Umeå in 2025, and during 2026, 23 additional sites, including all Swedish university hospitals, are preparing to launch – corresponding to ~50% of newly diagnosed prostate cancer patients in Sweden.

## Discussion and conclusion

The strength of this study lies in its broad ethical approval including several aspects and research questions in relation to precision medicine combined with a pragmatic participant inclusion procedure integrated into the clinical workflow, a biomarker workflow, automated follow-up data retrieval, and participants’ consent to be invited to additional clinical studies or trials based on collected information. In addition, the SPRINTR infrastructure provides a foundation for prostate cancer research, with shared administration, legal agreements, and IT systems. This creates an open research platform to validate and develop diagnostic and treatment-stratifying biomarkers, including AI tools.

Promising biomarkers must be carefully evaluated and compared with studies on quality of life and health economics. Precision-medicine biomarkers are often evaluated separately in unique settings. SPRINTR enables back-to-back analysis of different biomarkers in a prospective setting, along with health economic assessments across multiple disease stages. [14,15]

The expanding cohort with long-term follow up, imaging, and stored tissue enables diverse studies. Combined evaluation of MRIs, clinical variables, comorbidities, and tissue- or liquid based omics could support development of multimodal tools to predict early relapse and identify actionable targets.

SPRINTR aims to reduce inequality in patients’ access to information about and invitations to clinical trials. Its pragmatic, adaptable site-participation model, with varying involvement levels and a digitalized informed-consent process, enables integration into any diagnostic unit in Sweden. This increases patients’ chances of being contacted directly by the central study team based on eligibility, regardless of location, and reduces dependence on individual physicians in recruitment.

All Swedish Universities with medical faculties are part of SPRINTR, and all components are generic and designed for national reuse and scalability across cancer types. SPRINTR is aligned with national precision-medicine initiatives, including Genomic Medicine Sweden [14, 15], Clinical Trials Sweden (national clinical studies network) [16], Biobank Sweden (national biobanking infrastructure) [17], the Swedish Comprehensive Cancer Center network, and precision-medicine centers at university hospitals to achieve synergies molecular diagnostics, data solutions, and competence development. Thereby, SPRINTR aligns with emerging international developments in diagnosis-agnostic precision-medicine infrastructures, including initiatives such as Joint Action on Personalized Cancer Medicine (JA PCM), reflecting a broader transition toward platform-based models for clinical research and biomarker development. Dissemination of the SPRINTR concept will be shared within the EUnetCCC initiative [18].

In summary, the aim is to build a research infrastructure that integrates multimodal biomarker research to accelerate precision-medicine development and clinical implementation. By establishing a broad, well-documented, study-ready population, SPRINTR will increase the number of men invited to clinical studies and trials. SPRINTR supports prognostic and predictive research using multimodal datasets and secure follow-up. Ultimately, SPRINTR seeks to improve survival and quality of life for men with prostate cancer by advancing the clinical use of treatment-predictive biomarkers.
